# The *Streptococcus pyogenes* vaccine landscape

**DOI:** 10.1038/s41541-023-00609-x

**Published:** 2023-02-14

**Authors:** Donald R. Walkinshaw, Meghan E. E. Wright, Anne E. Mullin, Jean-Louis Excler, Jerome H. Kim, Andrew C. Steer

**Affiliations:** 1 Shift Health, Toronto, Canada; 2 International Vaccine Institute, Seoul, Republic of Korea; 3 Co-Chair, Strep A Vaccine Global Consortium (SAVAC) Executive Committee, Seoul, Republic of Korea; 4 Distinguished Visiting Professor, Seoul National University, Seoul, South Korea; 5 Murdoch Children’s Research Institute, Melbourne, Australia; 6 University of Melbourne, Melbourne, Australia

**Keywords:** Bacterial infection, Bacterial infection, Pharmaceutics

## Abstract

Recent efforts have re-invigorated the *Streptococcus pyogenes* (Group A Streptococcus) vaccine development field, though scientific, regulatory and commercial barriers persist, and the vaccine pipeline remains sparse. There is an ongoing need to accelerate all aspects of development to address the large global burden of disease caused by the pathogen. Building on over 100 years of *S. pyogenes* vaccine development, there are currently eight candidates on a product development track, including four M protein-based candidates and four candidates designed around non-M protein antigens. These candidates have demonstrated proof of concept for protection against *S. pyogenes* in preclinical models, one has demonstrated safety and immunogenicity in a Phase 1 trial and at least four others are poised to soon enter clinical trials. To maintain momentum, the Strep A Vaccine Global Consortium (SAVAC) was established to bring together experts to accelerate global *S. pyogenes* vaccine development. This article highlights the past, present and future of *S. pyogenes* vaccine development and emphasizes key priorities, and the role of SAVAC, in advancing the field.

## Introduction

There is a long history of vaccine development against *Streptococcus pyogenes*, commensurate to the large global burden of disease caused by the pathogen^1–3^. However, the path to a successful candidate has been impeded by scientific, regulatory, and commercial barriers. Over the past 5 years, efforts at vaccine development have been re-invigorated. At the 2018 71^st^ World Health Assembly, the need to prioritize a Strep A vaccine was recommended as an intervention that would effectively reduce the burden of rheumatic heart disease (RHD)^4^. The World Health Organization (WHO) and partners subsequently developed a research and development technology roadmap that has served as a valuable strategic guide for vaccine development^5^, and, in turn, the Strep A Vaccine Global Consortium (SAVAC) has attempted to action the roadmap^6^. The field has seen new investment, progress in the development of some existing vaccine candidates, and entry of new candidates to the pipeline. Nonetheless, the vaccine pipeline remains sparse, and there is an ongoing need to accelerate all aspects of vaccine development so that the compelling case for an effective vaccine can be met.

## Vaccine history

Vaccine development and clinical studies date back well over 100 years. Vaccine antigen approaches have included inactivated whole cell, scarlet fever toxin, M protein and other non-M protein antigens^7^. A major focus of development since 1940 has been the M protein, a key virulence factor of *S. pyogenes*. This approach began with whole M protein, then was refined to N-terminal polypeptides and C-repeat peptides. Tens of thousands of study participants received M protein vaccines prior to the 1960s, including children. *S. pyogenes* pharyngitis human challenge studies of purified M protein in the 1970s demonstrated efficacy of up to 89%, with no serious adverse events detected in vaccinees^8–10^.

Despite these promising results, vaccine development faced a major obstacle in 1979 when the US Food and Drug Administration instituted a federal regulation (21 CFR 610.19) that prohibited “Group A streptococcus organisms or their derivatives” from vaccines because “Group A streptococcal antigens in bacterial vaccines and antigens may induce dangerous tissue reactions in humans”^11^. The panel cited a study by Massell et al.^12^ that was uncontrolled and involved the administration of partially purified type 3 M protein vaccine to 21 healthy siblings of patients with rheumatic fever. Two of the vaccinees developed rheumatic fever and one developed possible rheumatic fever. While concerns about multiple aspects of this study have been raised, the 1979 FDA regulation had the unintentional consequence of impeding future development of *S. pyogenes* vaccines. In 2006 the FDA revoked Subpart 610.19^13^, and currently the FDA does not provide specific requirements for a *S. pyogenes* vaccine.

From 2006–2016, *S. pyogenes* vaccine development has progressed slowly, still impacted by the lingering effects of the 1979 ruling. The major area of clinical development was in M protein-based vaccine development, although important pre-clinical advances also occurred in non M protein vaccines, including group A streptococcal C5a peptidase and others^14^. Early-phase clinical trials in healthy adult volunteers of 4 vaccine candidates were conducted: a 6-valent N-terminal M protein vaccine, a 26-valent N-terminal M protein vaccine, a 30-valent N-terminal M protein vaccine and a conserved C-repeat region M protein vaccine^15–18^. No serious safety signals were detected in these trials, and encouraging immunogenicity data were observed for all vaccine candidates.

## Current pipeline

**Fig. 1:**
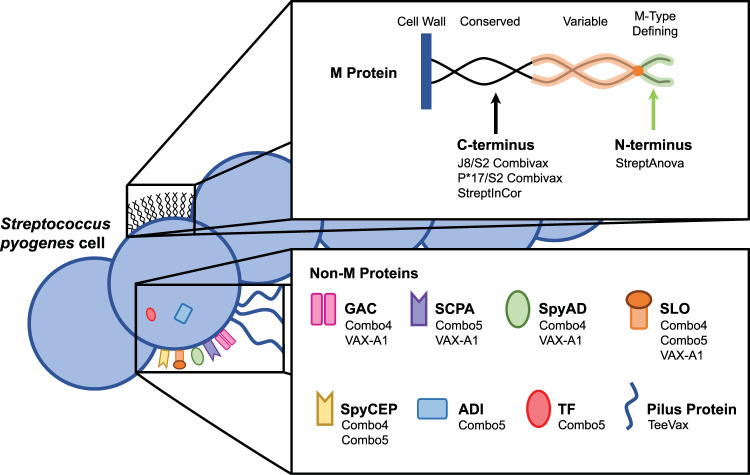
S. pyogenes antigens: Schematic of M protein and non-M protein antigens and corresponding vaccine candidates.

The current pipeline of *S. pyogenes* vaccine candidates on a product development track includes M protein-based concepts and candidates designed around non-M protein antigens. The following section provides an overview of both types of vaccine candidates, including their composition, theoretical coverage across *S. pyogenes* strains, demonstration of safety, immunogenicity and efficacy in various models and, where known, an indication of future development plans. Figure 1 presents a schematic of M protein and non-M protein antigens incorporated in the vaccine candidates of focus in this article.

### M protein-based candidates

Building on the long history of M protein-based vaccine research, several current *S. pyogenes* vaccine candidates have been designed around various M protein antigens (see summary of M protein-based candidates, Table 1). Given the high number of *emm* types and the hypervariability of M protein N-terminal regions, the only current vaccine candidate targeting N-terminal epitopes employs a multivalent approach (i.e. the 30-valent StreptAnova). Other M protein-based vaccines incorporate peptides from the more conserved C-terminus, and two of these (J8/S2 combivax and P*17/S2 combivax) combine a non-M protein (i.e. peptide from SpyCEP) in their formulation.

**Table 1:** *S. pyogenes* vaccine development pipeline: Overview of the most advanced M protein-based product development programs.

CANDIDATE	DEVELOPER	CURRENT DEVELOPMENT PHASE	ANTIGENS	ADJUVANT	THEORETICAL GLOBAL COVERAGE^a^	KEY REFERENCES
StreptAnova (30-valent)	University of Tennessee/ Vaxent	Phase 1a Completed 2020	Four protein subunits comprising the N-terminal regions of M proteins from 30 *S. pyogenes* serotypes	Aluminium hydroxide	48%^19^ Actual coverage may be higher (e.g. 80% in Africa) due to cross-opsonization^21^	^18,20^
J8/S2 combivax	Griffith University/ University of Alberta	Phase 1a *Ongoing*	J8 peptide from the M protein C-terminus combined with a 20-mer B-cell epitope (K4S2) from SpyCEP	Aluminium hydroxide	37%^19^ Actual coverage may be ~98% due to cross-reactivity of J8 alleles^25^	^17,23,26,27^
P*17/S2 combivax	Griffith University/ University of Alberta	Phase 1a *Ongoing*	P*17 peptide from the M protein C-terminus combined with a 20-mer B-cell epitope (K4S2) from SpyCEP	Aluminium hydroxide	37%^19^ Actual coverage may be ~98% due to cross-reactivity of J8 alleles^25^	^24,27^
StreptInCor	University of São Paulo	Preclinical	55-amino acid sequence peptide from the M5 protein conserved regions (C2 and C3)	Aluminium hydroxide	23%^19^ An earlier study showed 71% identity between StreptInCor and several emm types^30^	^29,31–33^

*SpyCEP* streptococcal interleukin-8 proteas.

^a^Based on antigen presence across sample of 2,083 *S. pyogenes* genomes^19^.

*StreptAnova*, developed by Dale et al. at the University of Tennessee (USA) with commercialization partner Vaxent, is an *emm*-type specific, adjuvanted (alum) vaccine with four protein subunits comprising the N-terminal regions of M proteins from 30 *S. pyogenes* serotypes. This candidate is the farthest along the development pathway, having completed a Phase 1a trial (in 2020) that demonstrated significant immunogenicity towards most of the targeted antigens^18^. Moreover, the trial showed that StreptAnova was well-tolerated and did not elicit autoimmune or cross-reactive antibodies^18^. Although theoretical coverage of StreptAnova across *S. pyogenes* strains based on a large-scale genomic analysis is 48% on a global level (and ranging from 28% in East Africa to 75% in North America)^19^, the StreptAnova developers have suggested actual coverage may be significantly higher. When accounting for the ability of StreptAnova to elicit cross-opsonic antibodies against non-vaccine serotypes^20^ and employing an *emm-cluster-based* *S. pyogenes* typing system, it has been proposed that StreptAnova could provide hypothetical coverage of 80.3% of isolates in Africa^21^. Additional clinical trials for StreptAnova are planned, including a Phase 2 efficacy study, pending funding^22^.

J8/S2 combivax and P*17/S2 combivax are related vaccines in development by Good et al. at Griffith University (Australia) and University of Alberta (Canada). Both vaccine candidates contain K4S2, a peptide with a modified B-cell epitope from *S. pyogenes* cell envelope proteinase (SpyCEP), combined with one of two versions of the p145 peptide from the M protein C-terminus: J8 for J8/S2 combivax^23^ and P*17 for its namesake candidate^24^. Both peptides in each candidate are conjugated to the CRM197 carrier protein. Although the J8 allele of the M protein is only found in 37% of *S. pyogenes* genomes^19^, both candidates are still expected to provide ~98% coverage across *S. pyogenes* strains because the J8 alleles cross-react immunologically^25^. In mouse studies, J8/S2 combivax and P*17/S2 combivax protected against skin and systemic infection from hypervirulent CovR/S strains of *S. pyogenes*^23^. The P*17/S2 combivax vaccine or prototype versions have also been shown to protect against upper respiratory tract infection in mice when formulated with alum, liposomes^26^ or with the liposomal adjuvant, CAF01^27^. An earlier version of the vaccine, J8-DT/Alum (which lacked the S2 peptide and in which diphtheria toxoid was used as the carrier protein), was shown to be immunogenic and safe following a single injection in a ‘pilot’ Phase 1 trial^17^. Approval has been granted by Health Canada (the Canadian Regulator) to undertake a Phase Ia trial of J8/S2 combivax and P*17/S2 combivax and the trial has begun. Upon success of the Phase 1a trial, the developers plan to advance the development of the lead candidate to a Phase 1/2 trial in Australia. The development pathway of the vaccine will likely involve a human challenge study in Australia in 2023^28^.

*StreptInCor*, from Guilherme et al. at the University of Sao Paulo (Brazil), is comprised of a 55-amino acid peptide from the M5 protein conserved regions (C2, C3) with B- and T-cell epitopes, adjuvanted with alum^29^. A large-scale genomic study showed that StreptInCor epitopes were found in 23% of *S. pyogenes* strains^19^, though an earlier study from the developers indicated that the StreptInCor sequence was 71% conserved amongst M protein sequences and that sequence differences do not affect opsonization, suggesting broad coverage^30^. In preclinical studies, StreptInCor has shown high levels of antigen-specific antibodies and survival against *S. pyogenes* infection challenge in mice as well as a lack of autoimmune reactions^29,31^. In minipigs, the candidate was well tolerated and displayed no harmful effects on heart tissue^32^. Studies in Wistar rats showed no evidence of toxicity after repeated intramuscular injections^33^. StreptInCor will be submitted to ANVISA (the Brazilian regulator) in early 2023 and the developer hopes to begin a clinical trial by the end of 2023^34^.

### Non-M protein-based candidates

Given the potential, but unproven, safety concerns of M protein-based vaccines, several *S. pyogenes* vaccine candidates are being designed around other antigens that provide broad coverage across *S. pyogenes* strains, and which have lower potential for cross-reactivity to host tissues (see summary of non-M protein-based candidates, Table 2). One of these antigens is Group A Carbohydrate (GAC), a surface polysaccharide comprising a poly-rhamnose backbone with an N-acetylglucosamine (GlcNAc) side chain. GAC is highly conserved and expressed in all *S. pyogenes* isolates^35^. Two groups have a vaccine candidate featuring GAC but each is using a different version of GAC and have conjugated their respective GAC antigens to different carrier proteins. The significance of these differences is yet to be fully understood, though preclinical results thus far indicate that both approaches may have their advantages (see below for details).

**Table 2:** *S. pyogenes* vaccine development pipeline: Overview of the most advanced non-M protein-based product development programs.

CANDIDATE	DEVELOPER	CURRENT DEVELOPMENT PHASE	ANTIGENS	ADJUVANT	THEORETICAL GLOBAL COVERAGE^a^	KEY REFERENCES
Combo4	GlaxoSmithKline/ GVGH	Preclinical	SpyCEP, SLO and SpyAD recombinant proteins and native GAC conjugated to CRM_197_ carrier protein	Aluminium hydroxide	>99%^19^	^41,43,44^
VAX-A1	Vaxcyte	Preclinical	SLO and SCPA recombinant proteins and modified GAC (Polyrhamnose) conjugated to SpyAD disease-specific carrier protein	Aluminium hydroxide	>99%^19^	^46^
Combo5	University of Queensland	Preclinical	Trigger factor (TF), inactivated versions of arginine deiminase (ADI), SLO, SpyCEP and SCPA	Squalene-in-water emulsion containing cholesterol (SMQ)	>99%^19^	^49,50,56^
TeeVax	University of Auckland	Preclinical	Multiple T-antigen domains from the pilus of the majority of S. pyogenes strains	Aluminium hydroxide	>95%^36^	^36,51^

*SpyCEP* streptococcal interleukin-8 protease, *SLO* streptolysin O, *SpyAD* putative surface exclusion protein, Spy0269, *GAC* Group A Carbohydrate, *SCPA* streptococcal C5a peptidase.

^a^Based on antigen presence across sample of 2,083 *S. pyogenes* genomes^19^.

Multiple *S. pyogenes* non-M protein antigens are also targeted by vaccine candidates. These proteins are highly conserved, being found in 95–99% of all characterized *S. pyogenes* isolates across the world^19,35,36^. Several of these proteins are noted below, with a brief description of their function. Streptolysin O is a secreted pore-forming toxin that is upregulated in virulent *S. pyogenes* isolates^35^. SpyCEP is a protease that drives immune evasion through cleavage of interleukin 8^37^. SpyAD is a surface-exposed adhesin that mediates *S. pyogenes* interaction with host cells^38^. Group A streptococcal C5a peptidase (SCPA) is an enzyme expressed on the cell envelope that mediates resistance to phagocytosis by cleaving the chemotaxin C5a on leukocytes^35^. Trigger factor (TF) is a peptidyl-prolyl cis-trans isomerase that is essential for the secretion and maturation of the *S. pyogenes* cysteine protease^39^. Arginine deiminase (ADI) is an enzyme that contributes to colonization and modulation of host immune response through conversion of arginine to citrulline and ammonia^40^. Finally, although variable, T-antigens collectively form a structurally conserved backbone of the *S. pyogenes* pilus, which is involved in adhesion, colonization and immune evasion^36^. Vaccine candidates combining subsets of these proteins, with or without combination with GAC, are described below.

*Combo4*, from GSK Vaccines Institute for Global Health (GVGH), GSK Vaccines (Italy), contains the native *S. pyogenes* GAC conjugated to the CRM_197_ carrier protein, SLO, SpyCEP and SpyAD^41^. GVGH has presented data indicating that the native GAC can induce a higher anti-GAC IgG response than poly-rhamnose and greater binding of anti-GAC antibodies compared to anti-poly-rhamnose antibodies to a panel of Strep A strains^42^. Preclinical studies of Combo4 adjuvanted with alum demonstrated immuno-protection in mouse models and efficacy in opsonophagocytic killing assays using sera from immunized rabbits^43,44^. GVGH is currently conducting GMP manufacturing and toxicity studies with Combo4 and is planning a Phase 1 dose-escalation study in Australia^45^.

*VAX-A1*, from Vaxcyte (USA), is based on work from the Nizet group at University of California, San Diego. VAX-A1 contains GAC^PR^, a modified version of GAC in which the GlcNAc side chain is removed, leaving the poly-rhamnose backbone^46^. In theory, GAC^PR^ may lower the risk of cross-immunogenicity compared to native GAC since the GlcNAc side chain on GAC has been implicated in provoking autoimmune cross-reactivity in RHD^46^. Moreover, the GAC^PR^ in Vax-A1 is conjugated to the *S. pyogenes* virulence factor SpyAD and this SpyAD-GAC^PR^ conjugate is combined with recombinant SLO and SPCA proteins and adjuvanted with alum^46^. Immunization of mice with VAX-A1 protected against *S. pyogenes* challenge in both a systemic infection model and localized skin infection model, with no observed signs of cross-reactivity to human heart or brain tissue epitopes^46^. Having initiated IND-enabling activities in late 2021, Vaxcyte is planning to provide guidance on expected timing for an IND application submission in the second half of 2022^47^.

*Combo5*, from Walker et al. at the University of Queensland (Australia), contains five recombinant proteins: SLO, SpyCEP, SCPA, TF and ADI, adjuvanted with SMQ (a squalene-in-water emulsion containing a toll-like receptor 4 agonist and QS21)^48^. In addition to offering broad coverage across *S. pyogenes* strains^48^, the vaccine candidate was designed to exclude *S. pyogenes* antigens potentially linked to autoimmune complications^49^. In earlier studies using alum as adjuvant, Combo5 reduced the severity of pharyngitis and tonsillitis but did not protect against colonization in NHP^49^; in mice, the candidate protected against superficial skin infections but not invasive disease^48,50^. In contrast, adjuvanting Combo5 with SMQ conferred protection against invasive challenge in mice, potentially owing to a more balanced Th1/Th2 immune response compared to Combo5 adjuvanted with alum, which produced a Th2-biased response^48^. Interestingly, Combo5/SMQ protected mice against invasive challenge in the absence of opsonizing antibodies, suggesting that an opsonizing antibody response may not be a correlate of protection for non-M protein vaccines^48^.

*TeeVax*, from Thomas Proft and Jacelyn Loh’s group at University of Auckland (New Zealand), is a multivalent vaccine targeting T-antigens, the major protein component of the surface-exposed *S. pyogenes* pili that are involved in adhesion and colonisation of the host during infection^36,51^. This vaccine candidate is comprised of three recombinant proteins (TeeVax1, TeeVax2 and TeeVax3), each consisting of a fusion of 6 unique T-antigen domains^36^. Combination of all three proteins (TeeVax1-3) elicited a robust antibody response in rabbits that was reactive to all 18 T-antigens included in the three proteins and was cross-reactive to the three remaining sub-types not included in any of the proteins^36^. This cross-reactivity to all 21 T-antigens would be expected to provide >95% *S. pyogenes* strain coverage^36^. Immunization with TeeVax1 adjuvanted with alum produced opsonophagocytic antibodies in rabbits and conferred protective efficacy against invasive disease in humanized plasminogen transgenic mice^36^. The developers are currently testing TeeVax with different adjuvants and plan to conduct analyses of humoral and cellular immune responses to TeeVax to gain further knowledge about correlates of protection^52^.

Together, these eight M protein and non-M protein vaccine candidates serve as a diverse, albeit small, set of concepts and approaches for continued and future clinical testing. Though it may be tempting to compare the candidates against one another in terms of theoretical likelihood of technical or regulatory success, our view is that such an exercise would be premature based on current knowledge. Correlates of protection for a vaccine against *S. pyogenes* remain unknown, animal models are not validated and standardized, and there has not yet been head-to-head comparison of any of the candidates. Thus, identifying optimal antigens and formulations for efficacy and safety requires further clinical trial testing, which the field is poised to embark on over the coming years.

## Future efforts

Despite a range of promising candidates, the pipeline of *S. pyogenes* vaccines remains relatively empty, especially when compared with pipelines for other infectious diseases that cause high global disease burden^53^. In an effort to attract more vaccine developers and investment to the field, SAVAC, established in 2019, has brought together *S. pyogenes* experts across multiple domains of expertise to fast track global *S. pyogenes* vaccine development^6^. SAVAC has highlighted the favourable cost effectiveness and return on investment of a *S. pyogenes* vaccine, as well as a series of key knowledge gaps. These gaps include: the scarcity of epidemiologic and economic data from low- and middle-income countries; incomplete understanding of measures of protection against *S. pyogenes* infection; the identification of immune correlates of protection against *S. pyogenes* infection, and the development of relevant new functional assays, the current absence of which represents a major impediment to vaccine development the identification of immune correlates of protection against *S. pyogenes infection*, and the development of relevant new functional assays still lacking and representing a major impediment to vaccine development^54^; the requirement for standardisation of safety surveillance; and the need for concerted advocacy efforts to raise the profile of the burden of *S. pyogenes* disease and how a vaccine could address this burden. The next phase of SAVAC’s work will be focused on filling these gaps by coordinating research to generate better burden of disease estimates, drawing together relevant stakeholders to establish guardrails for safety surveillance, boosting efforts around advocacy, and a range of other engagement activities. An innovative systematic framework outlining the properties of accurate and robust burden of disease data has been developed to guide vaccine development and evaluation and prioritize research and surveillance activities^3^. There is an opportunity to learn from the experience of vaccine development for SARS-CoV-2^53^, including the use of vaccine technologies such as mRNA^55^ and accelerated or adaptive clinical trial designs. As lead *S. pyogenes* vaccine candidates approach clinical trials, SAVAC is anticipating the steps necessary to support the field over the next five years. These steps include: 1) preparing for vaccine efficacy trials in low- and middle-income countries by gathering the epidemiological, economic and societal data that is currently lacking, while simultaneously strengthening surveillance and laboratory activities as well as clinical trial capacity through a network of sentinel sites in low- and middle-income countries; 2) preparing industry by engaging with vaccine developers and manufacturers to highlight the need and commercial case for a Strep A vaccine and understanding the barriers to vaccine development with a view to accelerating the Strep A vaccine pipeline; and 3) preparing key non-industry stakeholders by engaging with relevant non-industry stakeholders (e.g., WHO, global funders, national policy makers, NITAGs, experts in laboratory and safety surveillance) to address barriers and enhance implementation efforts for a future Strep A vaccine.

## Data Availability

Data sharing not applicable to this article as no datasets were generated or analysed during the current study.
